# MSCT Assessment of Perivascular Adipose Tissue and Visceral Fat Characteristics in Aortic, Iliac, and Lower Limb Aneurysms

**DOI:** 10.3390/biomedicines14061260

**Published:** 2026-05-31

**Authors:** Ante Pelivan, Benjamin Benzon, Danijela Budimir Mršić

**Affiliations:** 1 Department of Radiology, General Hospital of Šibenik—Knin County, Ul. Stjepana Radića 83, 22000 Šibenik, Croatia; ante.pelivan11@gmail.com; 2 School of Medicine, University of Split, Šoltanska 2, 21000 Split, Croatia; benjamin.benzon@mefst.hr; 3 Clinical Department of Diagnostic and Interventional Radiology, University Hospital Split, Spinčićeva 1, 21000 Split, Croatia

**Keywords:** PVAT, visceral fat, aneurysm, MSCT

## Abstract

**Background:** Perivascular adipose tissue (PVAT) plays a significant role in the atherosclerotic processes of arteries and aneurysmal occurrence and can be assessed by increased PVAT density in Hounsfield units (HU) on MSCT. Our objectives were to examine possible differences and associations of PVATs in several locations of aneurysmal occurrence (abdominal aorta, iliac, and lower extremity arteries), measured on the aneurysmal site and the same location on an unaffected artery. Also, we examined the possible association of aneurysmal PVAT with visceral fat parameters. **Methods:** In this retrospective and cross-sectional study, a total of 198 patients were included and divided into four groups: single abdominal aortic aneurysm (AAA, *n* = 55), single iliac artery aneurysm (*n* = 36), single lower extremity (leg) artery aneurysms of femoral or popliteal arteries (*n* = 43), and multiple aneurysms in one individual (AAA and iliac artery, *n* = 64). PVAT density measurements were performed at the level of the widest lumen of the aneurysm and at the same level on the contralateral artery. Aneurysmal diameter and thrombus width were also measured. The volume and the mean attenuation of visceral fat were automatically calculated by manually designating the targeted segmentation anatomical item. **Results:** A difference in PVAT densities among the first three groups of patients with a single aneurysm at different location was found (*p* = 0.027), as well as at the contralateral side (*p* < 0.001). In patients with multiple aneurysms, higher PVAT density was found at the iliac site compared to the AAA site (*p* = 0.002, paired test). Aneurysm diameter correlated to thrombus width, but not with PVAT densities. Comparison of PVAT densities with visceral fat densities showed moderate positive correlation (r = 0.446, Pearson correlation coefficient; ρ = 0.387, Spearman rank correlation; *p* ≤ 0.001), that was significant in the case of abdominal and iliac artery aneurysms, but not with leg aneurysms. **Conclusions:** Increased densities of PVAT were found on the aneurysmal site, suggesting its probable role in aneurysmal occurrence. PVAT density varied according to aneurysm location. Visceral fat may be associated with increased PVAT density and aneurysm development in abdominal and iliac regions, whereas this association was not observed in leg aneurysms.

## 1. Introduction

Aneurysms are focal pathological dilation of the arterial lumen. They are a common and important clinical entity due to the risk of arterial ruptures or thromboembolic complications. The most common aneurysms occur as fusiform abdominal aortic aneurysms (AAAs) and are defined as a dilation of the aortic lumen by more than 3 cm, or aortic diameter greater than 50% of its diameter at the level of the diaphragm [1,2]. The peripheral arterial aneurysms, particularly those involving the iliac, femoral and popliteal arteries, have an increasing incidence and clinical relevance. Peripheral aneurysms are defined as dilatation ≥ 1.5 times the normal caliber of the particular artery, and in the case of iliac arteries, a lumen ≥ 2.5 cm in diameter is a diagnostic criterium [3,4,5,6,7,8].

The main risk factors for the formation of aneurysms (especially AAA) include cigarette smoking, coronary artery disease, severe obesity, and a positive family history [9,10,11]. The multiple pathophysiological processes in the formation of AAA and aneurysms of peripheral arteries include an inflammatory response with consequent degradation of the extracellular matrix, various matrix metalloproteinases responsible for the destruction of elastin and collagen, oxidative stress, intramural thrombosis, and neovascularization [1,9,12,13,14,15]. In peripheral arteries (especially popliteal), in addition to atherosclerotic changes [16], the anatomical location in popliteal fossa and hemodynamic stress play an important role in the etiology of aneurysms [4]. In addition to the above, an important role is performed by perivascular adipose tissue (PVAT), which is located immediately adjacent to most blood vessels, except for cerebral vessels. In addition to its supporting role, it acts as a paracrine and endocrine organ that produces biologically active molecules [17,18,19,20]. In the physiological state, PVAT has anti-inflammatory properties. However, in conditions such as arterial hypertension, atherosclerosis, diabetes, and obesity, PVAT dysfunction occurs and the secretion of proinflammatory molecules is associated with the development of atherosclerotic plaques, vascular wall calcifications, and progression in the size of aneurysms [20,21]. There is also a difference in the composition and type of PVAT, so in the area of the thoracic aorta, there is predominantly brown adipose tissue; around the coronary arteries and the abdominal aorta is beige adipose tissue; and, according to the bifurcation of the aorta, around the iliac, femoral, mesenteric and carotid arteries is white adipose tissue, which is most susceptible to inflammatory processes [22]. Due to the development of the inflammatory process, an increase in the value of PVAT density in Hounsfield units (HU) on computed tomography (CT) sections is to be expected, which in previous studies has been shown to be a predictor of AAA progression [18,23,24]. When it comes to the impact of dysfunctional PVAT on peripheral arteries, there is one study by Fortunati et al., which showed a positive correlation between increased density of PVAT and the degree of stenosis of certain peripheral arteries. However, to our knowledge, peripheral aneurysms have not been studied in relation to their PVAT [25]. Visceral adipose tissue is a type of white adipose tissue accumulated in the abdominal cavity that is closely related to PVAT by sharing embryonic origin, accumulation, and function, and has been shown to contribute to the atherosclerotic process [26,27,28,29]. While most studies have focused on abdominal aortic PVAT and visceral adipose tissue [17,18,19,21,23,24], the relationship between histologically different PVATs of peripheral arteries and visceral adipose tissue is yet to be explored. We believe that understanding their relationships in anatomical regions other than the abdomen, such as the pelvis and lower extremity, could deepen our knowledge of overall cardiovascular risks.

Based on all of the above, the primary goals of our study were to examine possible differences in the PVAT density (measured in HU on CT scans) on the site of the aneurysm (abdominal, iliac and leg arteries) and at the same level on the unaffected contralateral artery. In addition, we investigated whether PVAT densities differ according to aneurysm location and size. Finally, we assessed the relationship between PVAT of peripheral arteries (iliac, femoral, and popliteal) and visceral fat volume, and compared these findings with those observed in abdominal aortic aneurysms. Due to the scarcity of research on PVAT in iliac and lower extremity arteries, our findings contribute important preliminary evidence and may provide a basis for future prospective investigations.

## 2. Materials and Methods

### 2.1. Study Design and Participants

**Figure 1:**
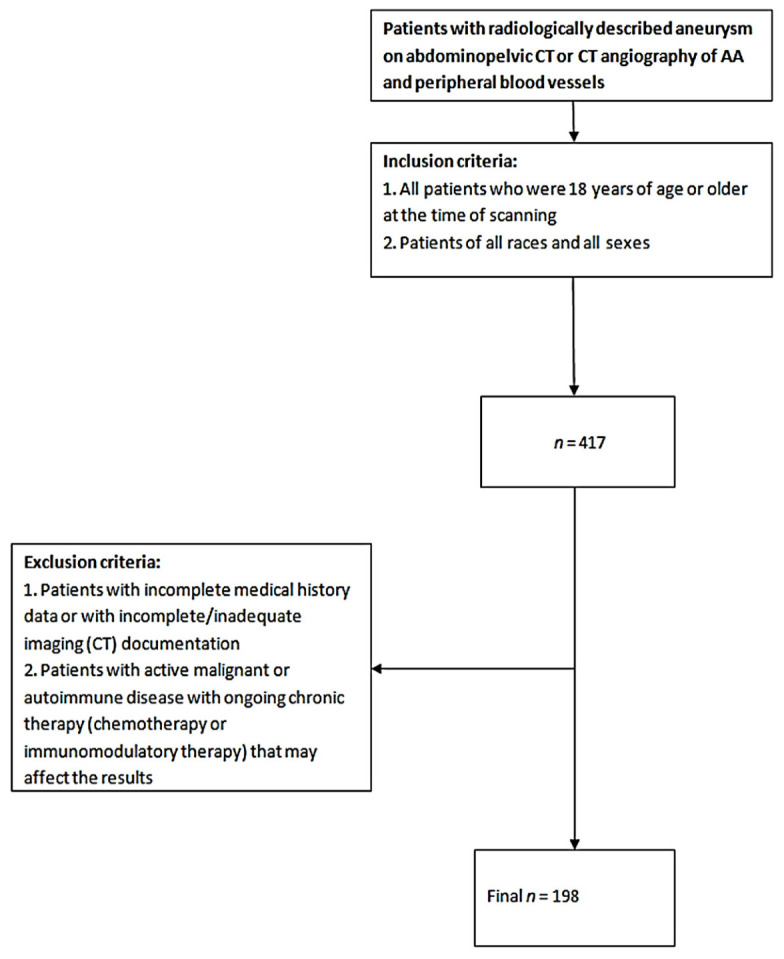
Flow chart of included study population.

The study is retrospective in its nature and cross-sectional design. It was conducted at the Clinical Department of Diagnostic and Interventional Radiology of University Hospital Center Split, an academic and tertiary medical hospital center. We retrospectively collected and analyzed all patients who underwent a postcontrast multislice CT of the abdomen and pelvis, as well as multislice CT angiography of the abdominal aorta and peripheral blood vessels, between 15 February 2021 and 23 October 2024. Included patients had radiologically described aneurysm of the abdominal aorta, iliac, or leg arteries, including the femoral and popliteal arteries. All patient medical history data were retrieved from the Hospital Information System (HIS). Inclusion criteria were: (1) all patients who were 18 years of age or older at the time of scanning; and (2) patients of all races and all sexes. Exclusion criteria were: (1) patients without complete medical history data or without complete/inadequate imaging documentation; and (2) patients with active malignant or autoimmune disease with ongoing chronic therapy (chemotherapy or immunomodulatory therapy) that may affect the results, which is shown in detail in Figure 1.

Of the total 417 subjects who met the inclusion criteria, after subsequent filtering according to the exclusion criteria (*n* = 219), a total of 198 patients were included in the study. The included patients were divided into four groups: the first group consisted of patients with abdominal aortic aneurysm (AAA; *n* = 55), the second group included those with iliac artery aneurysms (common iliac artery—CIA, internal iliac artery—IIA, and external iliac artery—EIA; *n* = 36), the third group consisted of patients with lower extremity/leg aneurysms (common femoral artery—CFA, superficial femoral artery—SFA, and popliteal artery—PA; *n* = 43), while the last group consisted of patients with multiple/combined AAA and iliac artery aneurysms (*n* = 64). The fourth group was heterogeneous; however, the analysis mainly focused on within-patient comparisons between AAA and iliac aneurysms. The lower extremity aneurysms observed in three patients were incidental and were not considered the primary focus of the analysis. This group allowed the assessment of valuable intraindividual differences in PVAT density across different aneurysm locations, which were not possible to examine with first three groups.

### 2.2. Analysis of Imaging Data

CT of the abdomen and pelvis, as well as CT angiography of the abdominal aorta and peripheral arteries, was performed on a 128-slice CT device Siemens Somatom Definition AS, Germany. The CT parameters used during imaging are tube current from 113 to 200 mAs and tube voltage of 120 kVp, with automatic value adjustment depending on the physical structure of the individual subject. Different contrast phases were performed on the CT scans based on individual requirements: non-contrast scan, early arterial phase (15–25 s after contrast agent injection), portal venous phase (70–90 s delay), and excretory phase (5–10 min delay). Of the 198 subjects included in the study, 185 had measurements performed in the arterial phase of the scan, eleven had non-contrast and venous phase of imaging (7 respondents in the first group, 2 in the second, and 2 in the fourth group). Measures were performed in the non-contrast phase. Two subjects had only the non-contrast scan (one each in the first and fourth groups).

**Figure 2:**
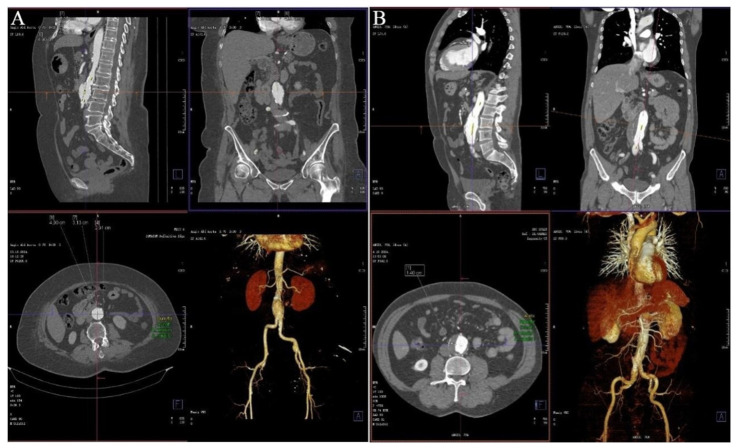
Sagittal, coronal, and axial sections of AAA show a 3D measurement of aneurysm size in millimeters (**A**). If a parietal thrombus is present, the width at its widest point on the axial section is measured (**B**).

The size of all aneurysms was measured in three dimensions (anteroposterior—AP × laterolateral—LL × craniocaudal—CC) and expressed in millimeters (mm). If present, the width of the parietal thrombus of the aneurysm at its widest point was also measured (width expressed in mm). A detailed overview of the described measurements is shown in Figure 2.

#### 2.2.1. PVAT Attenuation Analysis

**Figure 3:**
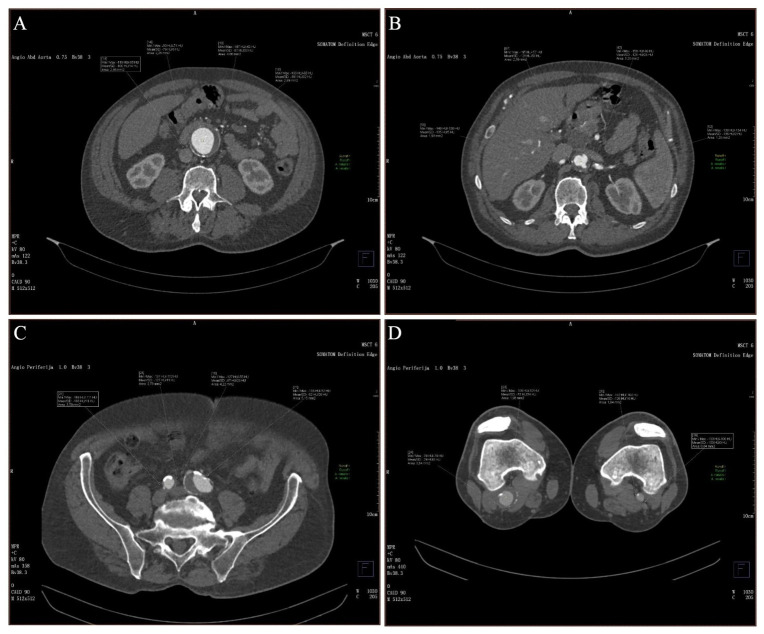
Axial CT scans show the method of measuring PVAT density. (**A**) At the widest part of the AAA, measurements were made at a distance of 2–5 mm and at 10 mm from the aortic wall on both sides. (**B**) As a control group, PVAT values in the AAA were measured at equal distances above or below the aneurysm. (**C**) In iliac artery aneurysms (left CIA aneurysm shown), PVAT attenuation values were measured at 2–5 mm from the vessel wall on both sides and, as a control group, attenuation values on the opposite side on the same CT scan when possible. (**D**) PVAT values were measured in the same way on peripheral arteries (right popliteal artery aneurysm shown).

PVAT attenuation/density values were measured in HU. In the AAA group of subjects, PVAT density measurements were performed at the level of the widest lumen of the aneurysm on the left and right anterior sides (imagining an aneurysm as a trigonometric circle, the measurements covered approximate areas at angles of 45° and 135°). Measurements were performed at two distances from the aortic wall, at 2–5 mm and at 10 mm, by drawing the line perpendicular to the blood vessel wall in the above-mentioned areas of surrounding fat tissue and then measuring circular regions of interest (ROI), i.e., reading the measured average attenuation value of the fat tissue. We did not perform measurements at a distance of less than 2 mm from the aortic wall in order to minimize the possible effect of the partial volume effect. Similarly, PVAT attenuation measurements at a distance of 10 mm from the aortic wall were performed for a more adequate and objective effect. When it came to AAA, as a control group of PVAT density values, we performed measurements at levels above or below the aneurysm, also within the aforementioned intervals of distance from the aortic wall. In iliac, femoral, and popliteal artery aneurysms, attenuation values of PVATs were also measured at the level of the widest lumen of the aneurysm, at a distance of 2–5 mm from the blood vessel wall on the left and right side as already mentioned, as far as the anatomical structures and position allowed. If there was an objective anatomical obstacle to measuring PVAT density at the specified distances at the level of the largest aneurysm diameter, then measurements were taken at the next closest section, cranial or caudal, to the widest part of the aneurysm though remaining adjacent to the aneurysmal zone. Control values were measured on the contralateral side without aneurysmal expansion, at the same distance from the vessel wall, on both sides. The PVAT density measurement method is shown in Figure 3. One radiologist with a decade of radiological experience performed the measurements after careful training and detailed methodological preparation.

After the measurements were performed, 10% of the subjects in each of the four groups were randomly selected, measurements of PVAT attenuation values, volume and density of visceral adipose tissue were repeated, and the Intraclass Correlation Coefficient (ICC) was calculated (Table A1).

#### 2.2.2. Analysis of Volume and Mean Attenuation Values of Visceral Adipose Tissue

**Figure 4:**
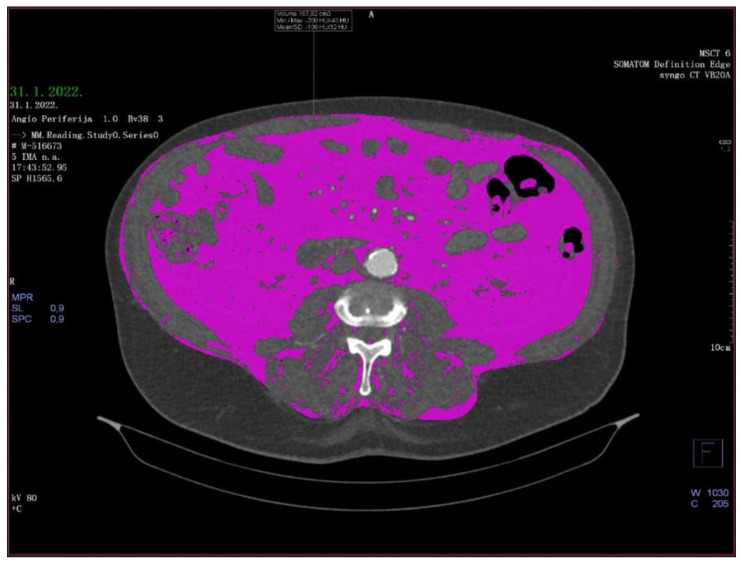
An example of volume measurements and mean attenuation values of visceral adipose tissue done by post-processing Siemens Syngo.via Anatomy Visualizer software VB60A HF08 software. Purple color denotes visceral fat content.

Siemens Syngo.via Anatomy Visualizer software VB60A HF08 was used to measure the volume and mean attenuation of visceral fat. All measurements were performed by manually marking the targeted segmentation anatomical object using the Anatomy Visualizer tools, which further automatically calculated the volume of visceral fat in cm^3^ and the mean attenuation of visceral fat in HU. The anatomical level for measuring the volume of visceral fat is the periumbilical region, i.e., the L3-L4 level [30]. The appearance of the measurement of the volume of visceral fat is shown in Figure 4.

#### 2.2.3. Statistical Analysis

Continuous variables were expressed as mean ± standard deviation (SD) and median with interquartile range (IQR). Categorical variables were reported as frequencies and percentages calculated from available data, with missing counts noted separately.

Cross-group comparisons of continuous and categorical variables were performed using the Kruskal–Wallis H test and Pearson’s chi-square test, respectively. Multivariable modeling was done with a linear regression mixed-effects model with random intercept. Analyses were performed using Python 3.12 with SciPy 1.14, NumPy 1.26, and pandas 2.2. Figures were generated with Matplotlib 3.9. Code for Python was generated by Claude Opus 4.6 [31].) A two-tailed *p* < 0.05 was considered significant, and Bonferroni correction was applied for multiple comparisons.

## 3. Results

A total of 198 participants were included in the study and were divided into four groups: the first three groups consisted of patients having a single aneurysm (AAA, iliac or lower extremity), while the fourth group consisted of patients having both AAA and iliac artery aneurysms. The AAA group had the highest median patient age, 73 years (range 50–91). Men constituted a significant majority in all groups (*p* < 0.001, Chi-square test), especially in the iliac aneurysm group, where they accounted for 94.4% of patients. Almost half of the patients were smokers (*p* = 0.028, Chi-square test), with the greatest proportion observed in the first group (71.4%). The most common comorbidity was arterial hypertension in all four groups (*p* = 0.011, Chi-square test). The highest median volume of visceral fat was found in the group with combined AAA and iliac artery aneurysms as 125.0 cm^3^, while the highest average value of visceral fat tissue density was found in the AAA group (−92.7 ± 8.7 HU). In the first group of patients, the median aneurysm diameter was 50 mm (range 30–86 mm), and this group also showed the highest median parietal thrombus width (16.4 ± 9.5 mm). PVAT densities were highest in patients with combined AAA and iliac artery aneurysms (−85.4 ± 10.3 HU), followed by AAA (−87.6 ± 13.3 HU) and lower extremity aneurysms (−89.5 ± 12.8 HU); ultimately, the lowest PVAT densities were recorded in patients with iliac artery aneurysms (−94.2 ± 15.9 HU), while the lowest mean values were observed in the control group of patients with AAA and iliac aneurysms (−128.3 ± 15.0 HU, *p* < 0.001). In the first group, PVAT densities measured at a distance of 10 mm from the vessel wall and averaged as −99.0 ± 14.1 HU (Table 1).

**Figure 5:**
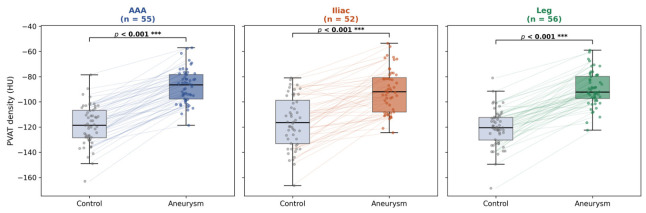
Paired PVAT density box plot: aneurysm vs. control sites.

Table 2 and Figure 5 demonstrated higher PVAT density at the arterial aneurysmal site in the first three patient groups compared with the control values of the contralateral vessel (*p* < 0.001). The difference ranged from +34.9 to +25 HU.

**Figure 6:**
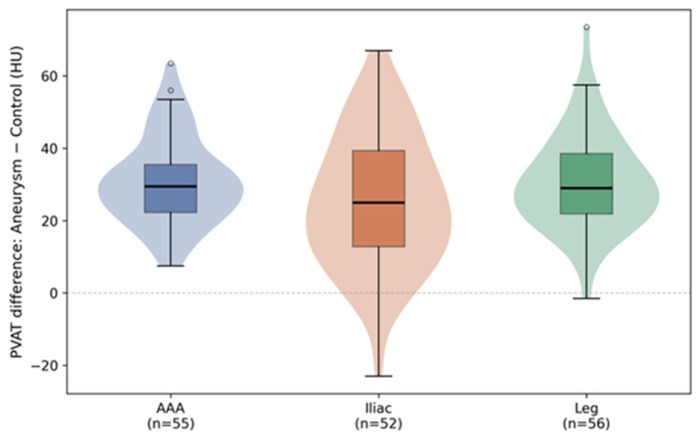
PVAT density elevation at aneurysm sites by location showed no significance (*p* = 0.082, Kruskal–Wallis test).

**Figure 7:**
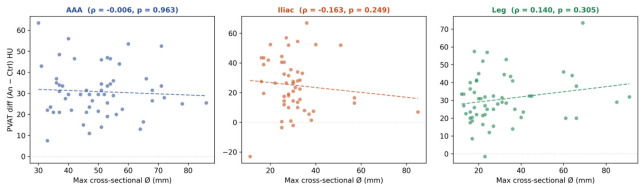
Comparison of cross-sectional aneurysmal diameters in the first three groups of participants having a single aneurysm with differences in PVAT densities measured along the aneurysm wall and at the contralateral site.

**Figure 8:**
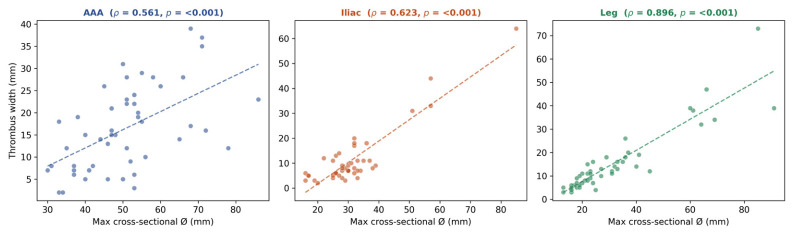
Positive correlation between cross-sectional aneurysmal diameter and parietal thrombus width in all three patient groups with a single aneurysm (AAA, iliac, or leg).

The difference in PVAT densities measured at the aneurysmal and control sites in participants in the first three groups was not significant (*p* = 0.082, Kruskal–Wallis test) with the highest median in the AAA group +29.5 HU, IQR [+22.2 to 35.5] (Table 3 and Figure 6). In the same groups of patients, there was no correlation between cross-sectional aneurysmal diameter and differences in PVAT densities (Figure 7); however, a moderate to strong correlation between aneurysm diameter and thrombus width was demonstrated, with correlation coefficients Rho ranging from 0.561 to 0.896, *p* <0.001 (Figure 8).

**Figure 9:**
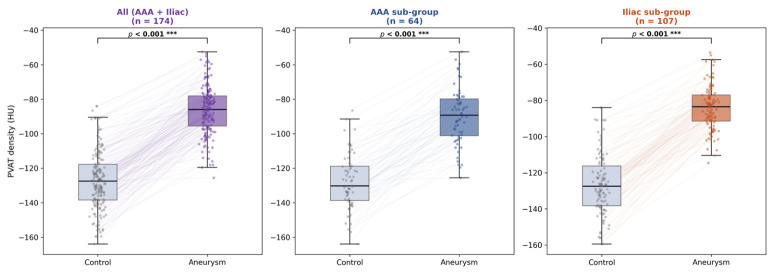
Comparison of PVAT density values measured at 2–5 mm from the artery wall showing higher PVAT values in the area of aneurysms in patients with multiple aneurysms, as well as in two subgroups (AAA and iliac subgroup); *n* = number of aneurysms.

The fourth group of participants, which includes AAA, iliac, and leg artery aneurysms, comprised 64 patients with a total of 174 aneurysms (64 AAA, 107 iliac aneurysms, and 3 incidental leg aneurysms) and showed a significant difference in the densities of aneurysmal PVAT compared to the control measurement site (Figure 9). Iliac arteries showed the highest mean PVAT density values in the area of the aneurysms (−84.0 ± 12.1 HU). In the control group, the mean value was −126.7 ± 17.1 HU, corresponding to a density difference of +42.6 ± 14.4 HU (Table 4).

**Figure 10:**
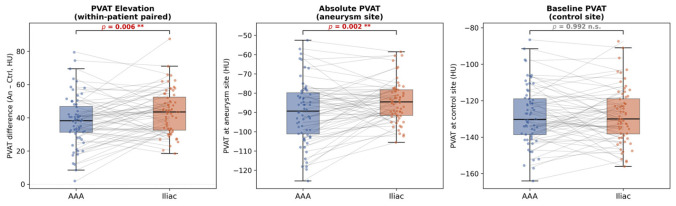
Differences in the mean PVAT density of AAA and iliac aneurysms within individual participants, showing higher PVAT density values within iliac arteries.

When comparing PVAT density values of AAA in relation to iliac artery aneurysms in each individual participant in the fourth group of patients, higher mean PVAT density values in both groups of arteries compared to the controls were observed. The mean PVAT density of iliac arteries with aneurysms was −84.4 ± 10.3 HU, while in the control group −127.9 ± 15.1 HU. Furthermore, the mean density value of PVAT with AAA was −89.8 ± 15.9 HU compared to the control group, which was −127.9 ± 16.0 HU. From the above, a higher increase in the density value at the location of the aneurysm of the iliac group was also observed in comparison with AAA (*p* = 0.002, paired test). When observing the differences in the increased PVAT density within an individual participant, it is worth noting the higher value in the iliac group (+43.5 ± 13.4 HU) compared to AAA (+38.1 ± 15.1 HU), (*p* = 0.006, paired test) (Table 5 and Figure 10).

**Figure 11:**
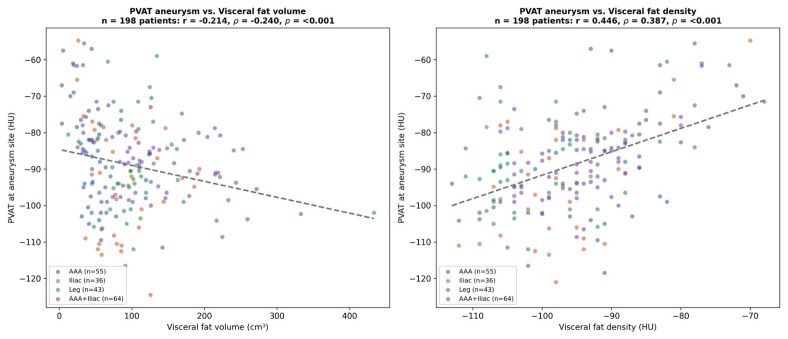
Display of PVAT density values measured at the aneurysm site in relation to visceral fat in all 198 participants included in the study.

**Figure 12:**
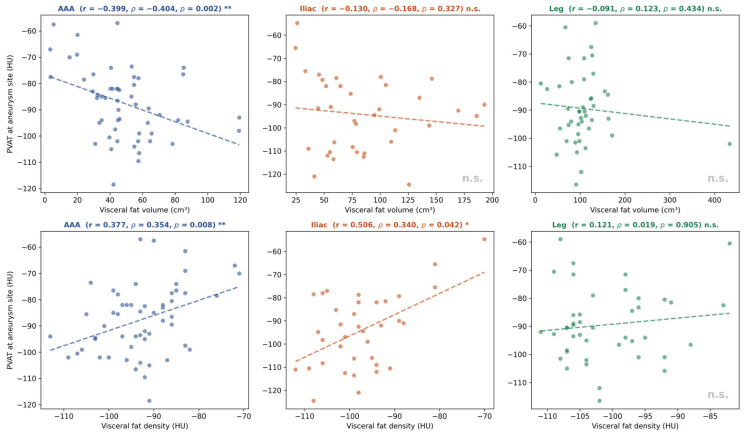
Graphic presentation of the comparison of PVAT density values with visceral fat tissue (volume—first line, density—second line) in the first three groups of participants.

Analysis of the relationship between the values of PVAT density at the aneurysmal site and visceral fat tissue values (volume in cm^3^ and density in HU) in all 198 participants divided into four groups, a weak negative correlation of PVAT with visceral fat volume was observed (r = −0.214, Pearson correlation coefficient; ρ = −0.240, Spearman rank correlation; *p* ≤ 0.001), and a mild to moderate positive correlation was observed with visceral fat density (r = 0.446, Pearson correlation coefficient; ρ = 0.387, Spearman rank correlation; *p* ≤ 0.001) (Figure 11). Further analysis of the participants in only the first three groups with a single aneurysm (AAA, iliac and leg) showed a positive correlation of PVAT density and visceral fat tissue density in patients with AAA (r = 0.377, Pearson correlation coefficient; ρ = 0.354, Spearman rank correlation; *p* = 0.008) and with iliac aneurysms (r = 0.506, Pearson correlation coefficient; ρ = 0.340, Spearman rank correlation; *p* ≤ 0.042), but not in patients with leg aneurysms. A moderate negative correlation between PVAT density and visceral fat volume (r = −0.399, Pearson correlation coefficient; ρ = −0.404, Spearman rank correlation; *p* = 0.002) should be singled out (Figure 12).

**Figure 13:**
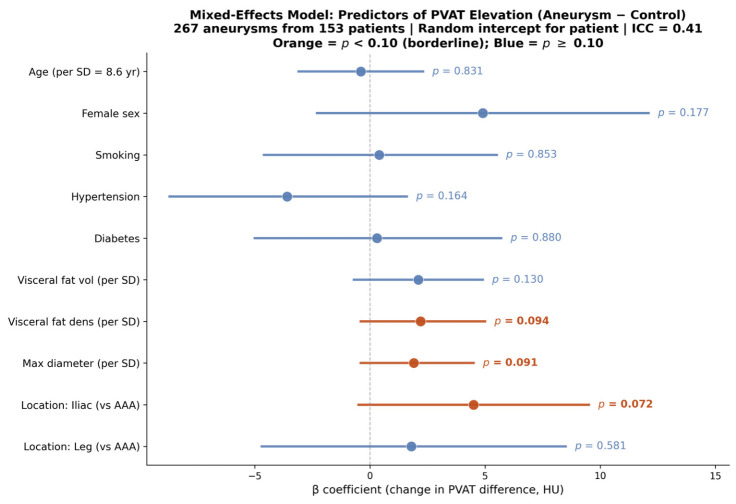
Graphical representation of a multivariable linear mixed-effects model.

In the multivariable linear mixed-effects model, the set determinants (age, gender, smoking, arterial hypertension, diabetes mellitus, visceral fat volume and density, maximum aneurysm diameter, and anatomical location) collectively explained 5.6% of the total variance in PVAT attenuation differences (marginal R^2^ = 0.056). The conditional R^2^, which incorporates the patient-level random intercept, was 47.0%, indicating that 41.4% of the variance is attributable to unmeasured between-patient factors not captured by clinical covariates (Figure 13).

## 4. Discussion

The results of the study showed higher PVAT density values along the aneurysmal wall compared to control contralateral site regardless of aneurysm location. No differences in PVAT densities among different aneurysmal locations (abdominal, iliac, leg) was found, except in the group of patients with multiple aneurysms, where a slightly higher average PVAT density values were observed in the iliac arteries compared to the abdominal aorta, suggesting the presence of intraindividual rather than interindividual differences. Comparing the perianeurysmal PVAT values with visceral fat tissue parameters, a positive correlation of visceral fat tissue densities in the groups with AAA and iliac aneurysms was observed, and the visceral fat effect was more prominent in the group of single AAA or iliac aneurysms, while it was not found in the group of leg aneurysms. Finally, the size of the parietal thrombus was associated with the aneurysmal size, but size was not associated with PVAT density.

Disturbance in homeostasis and dysfunction of PVAT, which is increasingly understood as a metabolically active tissue, leads to the development of inflammatory processes that have a significant impact on the blood vessel wall and, ultimately, to the progression of atherogenesis, with an increased risk of developing cardiovascular diseases. The aforementioned inflammatory processes in perivascular adipose tissue have been used as an imaging biomarker in the analysis of recorded CT scans, since it is assumed that adipose tissue altered by inflammation has higher HU values [17,22]. The results of our retrospective observational study showed an increase in PVAT density along the aneurysm wall in all examined groups of arteries, which is in accordance with certain previous studies [17,18]. Also, the majority of the subjects were male, while we determined smoking and arterial hypertension as relevant comorbidities. We did not confirm a positive correlation for other comorbidities such as diabetes mellitus and elevated cholesterol levels, and one of the significant reasons for this might be incomplete medical documentation. What sets our study apart from others is that we included aneurysms of iliac arteries, which to our knowledge were not part of similar trials, as well as aneurysms of leg arteries. The closest research we found by searching the literature is a study in which Fortunati et al. correlated elevated PVAT values with an increased degree of stenosis of peripheral arteries, but without analysis of aneurysms [25].

The differences in PVAT densities at different aneurysm positions (abdominal, iliac, and leg arteries) likely reflect the histological heterogeneity of perivascular adipose tissue along the examined vascular system as well as its intrinsic specific properties. Although all examined groups of arteries differ significantly in their anatomical position and hemodynamic characteristics, the increased attenuation of PVAT with aneurysms could indicate a common pathophysiological mechanism of the aneurysms themselves (degradation of the extracellular matrix, matrix metalloproteinases, and oxidative stress) and PVAT itself (which secretes biologically active molecules). Importantly, the significant differences observed between aneurysms and contralateral control positions suggest that changes in PVAT attenuation could be predominantly locally driven by inflammatory changes along the affected vessel segment. However, the magnitude of this response appears comparable across all arterial groups, which may explain the lack of intergroup statistical significance. In contrast, intra-individual analysis of patients in the fourth group with multiple aneurysms showed significantly higher attenuation values of PVAT in the area of iliac artery aneurysms compared to abdominal aortic aneurysms. This finding eliminates interindividual variability, which includes differences in systemic inflammation, metabolic status, and visceral adipose tissue, thus demonstrating subtle location-dependent effects of PVAT. Relatively higher PVAT density values with iliac artery aneurysms can be attributed to regional differences in adipose tissue with dominant white adipose tissue having the most pronounced proinflammatory activity [22] as well as local hemodynamic factors such as turbulent flow at the bifurcation of the artery. Ultimately, these findings suggest that although PVAT serves as a robust imaging biomarker of inflammatory changes associated with arterial aneurysms, its ability to discriminate between anatomical locations is limited in interindividual analyses but becomes apparent under controlled intraindividual conditions. By monitoring the difference in PVAT densities at the aneurysm site and at the control site with the diameter of the aneurysm itself, we did not record a significant positive correlation, and we consider the design of the study itself, which is retrospective, to be the reason for this. We believe that a positive and significant relationship could be confirmed if the subjects were prospectively followed with an increase in the number of subjects, as has already been established in previous studies [23].

A positive correlation of PVAT aneurysms of the aorta and iliac arteries with visceral fat densities was shown, showing more localized effects of visceral tissue (abdominal and iliac aneurysms) in comparison to leg aneurysms. The observed positive association between PVAT attenuation and VAT density with a negative association with VAT volume may be an indicator that fat composition and inflammatory status are more relevant than total adipose tissue volume. In healthy adipose tissue, large lipid-rich adipocytes have lower attenuation values (the attenuation threshold ranges from −190 to −30 HU), while in inflammation-altered adipose tissue, there are smaller adipocytes with an imbalance of fat and water content and edema of higher densities [32]. These structural changes and higher attenuation values are related to vascular inflammatory processes and represent a wide spectrum of remodeling and dysfunctional changes in adipose tissue [33]. Therefore, the differences in the volume and densities of VAT in our study become more understandable. A large volume of VAT may represent metabolically less active adipose tissue, while elevated VAT densities suggest proinflammatory remodeling, i.e., a biologically active component. It is also necessary to consider the regional aspect where the association between PVAT density and VAT in abdominal aortic and iliac artery aneurysms is observed, possibly due to anatomical proximity and microenvironmental signaling, where metabolically active VAT will enhance perivascular inflammation and aneurysm pathology. Such hypotheses that the quality of adipose tissue measured through attenuation on CT is a more important determinant of vascular inflammation than the volume of adipose tissue itself.

The multivariable linear mixed-effects model (Figure 13) shows that the examined demographic, clinical and imaging variables can explain only a minor part of the variability in PVAT attenuation differences (5.6%). A multivariable linear mixed-effects model shows the analysis of potential predictors of the increase in PVAT attenuation values between the aneurysmal and control artery segments. A total of 267 aneurysms in 153 patients were analyzed with a random intercept for each subject, where it was taken into account that some subjects have multiple aneurysms. The ICC value was 0.41 and showed that 41% of the total variability in PVAT differences can be explained by differences between the respondents themselves that are not covered by the measured variables. Most of the analyzed variables showed wide confidence intervals, which indicates the absence of an independent association with differences in PVAT densities. Age showed almost no effect on PVAT elevation (β close to 0; *p* = 0.831), suggesting that older age per se is not associated with more pronounced local inflammatory changes in PVAT. On the other hand, the results related to visceral fat tissue, aneurysm size, and anatomical localization are interesting. The average attenuation values of visceral fat tissue (β positive direction; *p* = 0.094) and the maximum diameter of the aneurysm (*p* = 0.091) may indicate a certain positive association with increased PVAT attenuation. However, none of the results reached definitive statistical significance. Perhaps the closest to this is the anatomical location, or more precisely the analyzed iliac aneurysms, which showed greater differences in the densities of PVAT compared to AAA (*p* = 0.072). Overall, the model shows that none of the single examined parameters fully explains the alterations of PVAT. These findings support the concept of PVAT as a dynamic imaging biomarker of vascular inflammation reflecting both local and systemic mechanisms involved in aneurysmal disease.

Despite the partially promising results of our study, in addition to the limitations and shortcomings already mentioned, the relatively small number of respondents may have an impact on the objectivity of the results. Also, we believe that it is very important to highlight the retrospective cross-sectional design of the study, which does not allow causal inference or assessment of temporal relationships between PVAT attenuation and aneurysm development. The examined groups are clinically and anatomically heterogeneous, especially the last group of respondents, which limits the comparison of results along the vascular territories. The included patient cohort reflects a real-world clinical population, in which the disease is predominantly observed in elderly male patients, particularly those with a history of smoking and arterial hypertension, among whom the condition is epidemiologically most prevalent. In this context, a complete elimination of gender bias was not methodologically feasible without compromising the representativeness of the sample. Furthermore, the control measurements differ depending on the location of the aneurysm, such as the non-aneurysmal segment in the AAA and the contralateral vessels in the peripheral arteries, which reduces the biological and methodological equivalence between the control groups. As one of the important limitations of the study, the measurements of PVAT attenuation values were performed manually using ROI sampling, which could lead to operator-dependent variability despite ROI standardization and high intraclass correlation coefficients, especially in anatomically constrained peripheral regions. When it comes to measuring the volume and average density of visceral adipose tissue, we must emphasize that although the calculation process is automatic, manual pre-process marking and processing leaves the possibility of variation between operators. Moreover, since CT acquisition phases were determined by clinical indication and not by a standardized study protocol and were retrospectively collected and analyzed, it is not possible to completely exclude variability in attenuation value measurements depending on contrast timing. Finally, despite the multivariable analyses conducted, confounding from demographic, metabolic, and vascular factors cannot be completely excluded.

As a way of verifying and attempting to objectify the manual measurements, 10% of the subjects in each group were randomly selected and the measurements were repeated, with the results shown in Table A1. The intraclass correlation coefficient analysis demonstrated excellent intra-rater reliability for nearly all CT-derived measurements, particularly for PVAT attenuation values, with ICC(3,1) values ranging from 0.953 to 0.999 for most PVAT measurements and very low coefficients of variation. It is important to note that the MDC_95_ values for PVAT attenuation at 2–5 mm were substantially lower than the observed aneurysm–control differences, indicating that the detected PVAT changes markedly exceeded measurement error and therefore likely reflect biological variation rather than technical variability. When it comes to the performed visceral fat tissue measurements, a few results showed slightly lower reproducibility (good rather than excellent ICC values), but the overall reliability analysis confirms the consistency of the manual ROI-based methodology used in this study.

Nevertheless, as a reinforcement of the objectivity of the measurement, and thus of the study itself, we believe that the use of AI in future research would contribute to the uniformity of the approach and more precise results, as shown in the work of Ginzburg et al. However, the main strength of our study is that this was the first study to investigate all locations of aneurysms from the abdominal aorta to the leg periphery and their relationship with PVAT, as well as the interrelationship with visceral adipose tissue, which was not done before. In this way, it may serve as an incentive for future research with the inclusion of more subjects, a prospective design that would include even more parameters, all with the aim of improving diagnostic outcomes.

## 5. Conclusions

In conclusion, this study found an increase in PVAT density among different aneurysmal locations, with the contralateral (control) site of the arteries in the first three group of patients having a single aneurysm, and in an intraindividual study of multiple/combined abdominal aortic and iliac artery aneurysms. PVAT values with iliac aneurysms showed higher densities. We also demonstrated a correlation between PVAT and visceral fat density in AAA and iliac aneurysms but not in leg arteries, showing no probable distant effect of visceral fat deposits.

## Figures and Tables

**Table 1:** Patient-level descriptive statistics by aneurysm location group. Values are presented as mean ± SD, median [range], or *n*/*N* (%). PVAT = perivascular adipose tissue; HU = Hounsfield units; Ø = diameter; An = aneurysm; Ctrl = control; Max cross-sect. = cross-sectional diameter (AP, LL). Patient-level values are averaged across multiple aneurysms where applicable.

Variable	AAA (*n* = 55)	Iliac (*n* = 36)	Lower Extremity (*n* = 43)	AAA + Iliac (*n* = 64)	*p*
**Demographics**					
Patients, *n*	55	36	43	64	
Age (years), mean ± SD	71.2 ± 8.9	72.7 ± 8.9	70.6 ± 9.6	70.9 ± 8.3	0.809 ^a^
Age, median [range]	73 [50–91]	72 [57–96]	71 [54–91]	72 [54–97]	
Male sex, *n* (%)	38 (69.1)	34 (94.4)	40 (93.0)	59 (92.2)	**<0.001** ^b^
**Comorbidities**					
Smoking, *n*/*N* (%)	30/42 (71.4)	12/26 (46.2)	16/36 (44.4)	23/53 (43.4)	**0.028** ^b^
*Missing*, *n*	13	10	7	11	
Arterial hypertension, *n*/*N* (%)	38/48 (79.2)	28/30 (93.3)	24/37 (64.9)	37/58 (63.8)	**0.011** ^b^
*Missing*, *n*	7	6	6	6	
Diabetes mellitus, *n*/*N* (%)	12/45 (26.7)	11/30 (36.7)	9/37 (24.3)	15/58 (25.9)	0.673 ^b^
*Missing*, *n*	10	6	6	6	
Cholesterol (mmol/L), mean ± SD	4.57 ± 0.99	3.91 ± 0.98	4.06 ± 1.24	4.34 ± 1.89	0.266 ^a^
*Available*, *n*	19	12	14	27	
**Visceral Fat**					
Volume (cm^3^), mean ± SD	48.7 ± 23.7	84.3 ± 44.1	108.4 ± 61.0	136.7 ± 72.8	**<0.001** ^a^
Volume (cm^3^), median	44.6	76.2	102.1	125.0	
Density (HU), mean ± SD	−92.7 ± 8.7	−97.5 ± 8.8	−101.0 ± 7.1	−93.3 ± 9.4	**<0.001** ^a^
**Aneurysm Characteristics**					
Aneurysms per patient, mean ± SD	1.0 ± 0.0	1.4 ± 0.7	1.3 ± 0.5	2.7 ± 0.9	**<0.001** ^a^
Max cross-sect. Ø (mm), mean ± SD	50.0 ± 12.7	31.6 ± 11.9	30.5 ± 17.6	39.3 ± 7.2	**<0.001** ^a^
Max cross-sect. Ø (mm), median [range]	50 [30–86]	30 [16–85]	23 [12–91]	39 [27–54]	
Thrombus width (mm), mean ± SD	16.4 ± 9.5	11.6 ± 11.8	15.1 ± 11.8	13.6 ± 6.2	**0.006** ^a^
*Available/Missing*, *n*	49/6	33/3	37/6	63/1	
**PVAT Density at 2–5 mm (HU)**					
Aneurysm site, mean ± SD	−87.6 ± 13.3	−94.2 ± 15.9	−89.5 ± 12.8	−85.4 ± 10.3	**0.027** ^a^
Control site, mean ± SD	−118.4 ± 15.7	−119.2 ± 18.3	−119.1 ± 12.9	−128.3 ± 15.0	**<0.001** ^a^
Difference (An − Ctrl), mean ± SD	+30.8 ± 11.9	+24.9 ± 15.9	+29.6 ± 11.2	+42.9 ± 13.2	**<0.001** ^a^
Difference, median	+29.5	+25.8	+29.0	+42.5	
**PVAT Density at 10 mm (HU)—AAA only**					
Aneurysm site, mean ± SD	−99.0 ± 14.1	—	—	—	
Control site, mean ± SD	−134.0 ± 17.5	—	—	—	
Difference (An − Ctrl), mean ± SD	+34.9 ± 16.2	—	—	—	

^a^ Kruskal–Wallis test; ^b^ Chi-square test. Significant *p*-values (<0.05) are shown in bold red. AAA = abdominal aortic aneurysm; Iliac = a. iliaca communis, externa, interna; Leg = a. femoralis communis, superficialis, poplitea; AAA + Iliac = patients with concurrent aortic and iliac aneurysms. An − Ctrl= Aneurysm − Control. Smoking, hypertension, and diabetes percentages calculated from available data; missing counts reported separately.

**Table 2:** The difference in PVAT densities measured at the aneurysmal site and control values of the contralateral vessel in the first three groups of patients with a single aneurysm.

Group	PVAT Aneurism (HU)	PVAT Control (HU)	Diff ± SD	Paired t	Wilcoxon	Cohen’s d
AAA (*n* = 55)	−87.6 ± 13.2	−118.4 ± 15.5	+30.8 ± 11.8	<0.001	<0.001	2.62
Iliac (*n* = 52)	−91.3 ± 17.1	−116.3 ± 20.2	+25.0 ± 18.3	<0.001	<0.001	1.37
Leg (*n* = 56)	−89.8 ± 13.3	−120.5 ± 14.6	+30.7 ± 13.2	<0.001	<0.001	2.32
AAA 10 mm (*n* = 54)	−99.2 ± 14.1	−134.0 ± 17.4	+34.9 ± 16.0	<0.001	<0.001	2.18

AAA = abdominal aortic aneurysm; Iliac = a. iliaca communis, externa, interna; Leg = a. femoralis communis, superficialis, poplitea; *n* = number of aneurysms; PVAT = perivascular adipose tissue.

**Table 3:** Median values of the difference in density of PVAT aneurysms and control groups in the first three categories of respondents.

	Median PVAT Difference (Aneurysm–Control) at 2–5 mm
AAA (*n* = 55)	+29.5 HU, IQR [+22.2 to 35.5]
Iliac (*n* = 52)	+25.0 HU, IQR [+12.8 to 39.4]
Leg (*n* = 56)	+29.0 HU, IQR [+21.9 to 38.5]

AAA = abdominal aortic aneurysm; Iliac = a. iliaca communis, externa, interna; Leg = a. femoralis communis, superficialis, poplitea; *n* = number of aneurysms; PVAT = perivascular adipose tissue.

**Table 4:** Within-patient comparison of mean PVAT density values at the aneurysmal site and at the control position in the fourth group of participants with multiple aneurysms, as well as in AAA and iliac aneurysm subgroups.

Subgroup	PVAT An (HU)	PVAT Ctrl (HU)	Diff ± SD	Paired t	Wilcoxon	d
All (*n* = 174, 64 pts)	−86.2 ± 13.9	−127.0 ± 16.6	+40.8 ± 14.8	<0.001	<0.001	2.75
AAA (*n* = 64, 64 pts)	−89.8 ± 15.9	−127.9 ± 16.0	+38.1 ± 15.1	<0.001	<0.001	2.52
Iliac (*n* = 107, 64 pts)	−84.0 ± 12.1	−126.7 ± 17.1	+42.6 ± 14.4	<0.001	<0.001	2.97

AAA = abdominal aortic aneurysm; Iliac = a. iliaca communis, externa, interna; *n* = number of aneurysms; PVAT = perivascular adipose tissue.

**Table 5:** Within-patient comparison of PVAT density in AAA and iliac aneurysms (fourth group with multiple aneurysms).

	AAA	Iliac	Paired Test P	Wilcoxon P
PVAT diff (An − Ctrl), HU	+38.1 ± 15.1	+43.5 ± 13.4	0.006	0.008
PVAT aneurysm site, HU	−89.8 ± 15.9	−84.4 ± 10.3	0.002	0.002
PVAT control site, HU	−127.9 ± 16.0	−127.9 ± 15.1	0.992	0.989

AAA = abdominal aortic aneurysm; Iliac = a. iliaca communis, externa, interna; PVAT = perivascular adipose tissue.

## Data Availability

The datasets are available upon request to the corresponding author.

## Appendix A

**Table A1:** Intra-rater test–retest reliability of CT measurements, measured by a single rater from two sessions on a 10% random sample. ICC(3,1) = two-way mixed model for consistency of single measures; SEM = standard error of measurement (√MSerror); MDC_95_ = minimal detectable change at 95% confidence (2.77 × SEM); CV = within-subject coefficient of variation; Bias = mean difference (measurement 1–measurement 2); LoA = Bland–Altman 95% limits of agreement; P^b^ = paired *t*-test for systematic bias.

Variable	*n*	ICC(3,1)	95% CI	Rating	SEM	MDC_95_	CV%	Bias	SD	95% LoA	P^b^
PVAT Density Measurements											
PVAT aneurysm (2–5 mm)											
AAA (*n* = 6)	6	0.998	[0.983, 1.000]	Excellent	0.92	2.53	1.0	−0.75	1.29	[−3.3, 1.8]	0.215
Iliac (*n* = 4)	4	0.999	[0.978, 1.000]	Excellent	0.61	1.70	0.6	−0.25	0.87	[−1.9, 1.4]	0.604
Leg (*n* = 4)	4	0.992	[0.884, 0.999]	Excellent	0.87	2.40	0.9	+0.50	1.22	[−1.9, 2.9]	0.474
AAA + Iliac (*n* = 12)	12	0.998	[0.993, 0.999]	Excellent	0.79	2.19	1.0	−0.46	1.12	[−2.6, 1.7]	0.183
PVAT control (2–5 mm)											
AAA (*n* = 6)	6	0.953	[0.705, 0.993]	Excellent	2.18	6.03	1.7	+1.25	3.08	[−4.8, 7.3]	0.366
Iliac (*n* = 4)	4	0.986	[0.808, 0.999]	Excellent	1.29	3.58	1.1	+2.50	1.83	[−1.1, 6.1]	0.071
Leg (*n* = 4)	4	0.999	[0.982, 1.000]	Excellent	0.34	0.94	0.3	+1.38	0.48	[+0.4, 2.3]	0.010
AAA + Iliac (*n* = 12)	12	0.999	[0.996, 1.000]	Excellent	0.66	1.83	0.5	+0.38	0.93	[−1.5, 2.2]	0.191
PVAT aneurysm (10 mm)											
AAA only (*n* = 6)	6	0.996	[0.969, 0.999]	Excellent	1.13	3.12	1.2	+0.92	1.59	[−2.2, 4.0]	0.218
PVAT control (10 mm)											
AAA only (*n* = 6)	6	0.960	[0.745, 0.994]	Excellent	3.28	9.08	2.3	+0.75	4.63	[−8.3, 9.8]	0.708
Aneurysm Dimensions											
AP diameter (mm)											
AAA	6	0.999	[0.991, 1.000]	Excellent	0.37	1.01	0.9	+0.33	0.52	[−0.7, 1.3]	0.175
Iliac	4	0.996	[0.938, 1.000]	Excellent	0.35	0.98	1.1	+0.25	0.50	[−0.7, 1.2]	0.391
Leg	4	1.000	—	Excellent	0.00	0.00	0.00	0.00	0.00	—	—
AAA + Iliac	12	0.999	[0.998, 1.000]	Excellent	0.32	0.89	0.9	+0.25	0.45	[−0.6, 1.1]	0.082
LL diameter (mm)											
AAA	6	0.999	[0.994, 1.000]	Excellent	0.39	1.07	0.9	+0.50	0.55	[−0.6, 1.6]	0.076
Iliac	4	1.000	—	Excellent	0.00	0.00	0.00	0.00	0.00	—	—
Leg	4	1.000	—	Excellent	0.00	0.00	0.00	0.00	0.00	—	—
AAA + Iliac	12	0.999	[0.998, 1.000]	Excellent	0.28	0.76	0.8	+0.17	0.39	[−0.6, 0.9]	0.166
CC length (mm)											
AAA	6	0.998	[0.986, 1.000]	Excellent	0.73	2.02	1.2	+2.33	1.03	[+0.3, 4.4]	0.003
Iliac	4	0.956	[0.483, 0.997]	Excellent	1.00	2.77	2.7	+2.00	1.41	[−0.8, 4.8]	0.066
Leg	4	0.995	[0.931, 1.000]	Excellent	0.58	1.60	1.5	+2.00	0.82	[+0.4, 3.6]	0.016
AAA + Iliac	12	0.997	[0.988, 0.999]	Excellent	1.16	3.22	2.5	+1.17	1.64	[−2.1, 4.4]	0.032
Thrombus width (mm)											
All sheets	26	1.000	—	Excellent	0.00	0.00	0.00	0.00	0.00	—	—
Visceral Fat Parameters											
Visceral fat volume (cm^3^)											
AAA	6	0.996	[0.973, 0.999]	Excellent	1.55	4.28	2.7	−1.80	2.19	[−6.1, 2.5]	0.099
Iliac	4	0.990	[0.860, 0.999]	Excellent	6.83	18.91	6.5	+1.98	9.65	[−16.9, 20.9]	0.709
Leg	4	0.929	[0.276, 0.995]	Excellent	3.93	10.87	3.7	+3.35	5.55	[−7.5, 14.2]	0.314
AAA + Iliac	12	0.997	[0.989, 0.999]	Excellent	2.22	6.15	2.8	+6.99	3.14	[+0.8, 13.1]	<0.001
Visceral fat density (HU)											
AAA	6	0.924	[0.557, 0.989]	Excellent	1.65	4.58	1.8	+1.67	2.34	[−2.9, 6.2]	0.141
Iliac	4	0.938	[0.342, 0.996]	Excellent	1.47	4.08	1.5	+4.50	2.08	[+0.4, 8.6]	0.023
Leg	4	0.854	[−0.099, 0.990]	Good	1.21	3.35	1.1	−0.25	1.71	[−3.6, 3.1]	0.789
AAA + Iliac	12	0.865	[0.598, 0.959]	Good	3.90	10.80	4.4	+3.33	5.52	[−7.5, 14.1]	0.060

ICC interpretation: excellent ≥ 0.90, good 0.75–0.89, moderate 0.50–0.74, poor and less than 0.50) [34]. The mean MDC_95_ for PVAT density at 2–5 mm was 2.65 HU, which is 9–15× smaller than the observed aneurysm–control differences (25–41 HU), confirming that the study findings substantially exceed measurement error.
